# Language reorganization patterns in global aphasia–evidence from fNIRS

**DOI:** 10.3389/fneur.2022.1025384

**Published:** 2023-01-06

**Authors:** Haozheng Li, Jianju Liu, Shan Tian, Shunjuan Fan, Tingwei Wang, Hong Qian, Gang Liu, Yulian Zhu, Yi Wu, Ruiping Hu

**Affiliations:** ^1^School of Rehabilitation Science, Shanghai University of Traditional Chinese Medicine, Shanghai, China; ^2^Department of Rehabilitation Medicine, Huashan Hospital, Fudan University, Shanghai, China; ^3^Department of Rehabilitation Medicine, Shanghai Fifth Rehabilitation Hospital, Shanghai, China

**Keywords:** global aphasia, fNIRS, picture naming, repetition phrase, left SMG

## Abstract

**Background:**

Exploring the brain reorganization patterns associated with language recovery would promote the treatment of global aphasia. While functional near-infrared spectroscopy (fNIRS) has been widely used in the study of speech and language impairment, its application in the field of global aphasia is still limited.

**Aims:**

We aimed to identify cortical activation patterns of patients with global aphasia during naming and repetition tasks.

**Methods and procedures:**

We recruited patients with post-stroke aphasia from the Department of Rehabilitation Medicine at Huashan Hospital. These individuals were diagnosed with global aphasia without cognitive impairments, as assessed by speech-language pathology evaluations. Age- and sex-matched healthy controls were recruited from the greater Shanghai area. During fNIRS measurement, patients and healthy controls completed the picture-naming and phrase repetition task. Cortical activation patterns on each of these language tasks were then compared between groups.

**Outcomes and results:**

A total of nine patients with global aphasia and 14 healthy controls were included in this study. Compared with the healthy subjects, patients with global aphasia showed increased activation in the left Broca's area, middle temporal gyrus (MTG), superior temporal gyrus (STG), and pre-motor and supplementary motor cortex (SMA) (*p* < 0.05) in the picture-naming task. Furthermore, the latency of the oxyhemoglobin (HbO) concentration in the left supramarginal gyrus (SMG) region had a strong negative correlation with their score of the naming task (*p* < 0.01). In the phrase repetition task, decreased activation was detected in the left SMA and SMG (*p* < 0.05) of patients relative to controls.

**Conclusion:**

The left SMG plays a critical role in the language function of patients with global aphasia, especially in their abilities to name and repeat. fNIRS is a promising approach to revealing the changes in brain activities in patients with aphasia, and we believe it will contribute to a deeper understanding of the neurological mechanisms and the establishment of a novel treatment approach for global aphasia.

## Introduction

Aphasia is a common functional disorder following a stroke and affects about 24–50% of all stroke survivors (1). In an observational study, global aphasia was the most commonly observed aphasia (33.33%) (2). Global aphasia is the most severe form of post-stroke aphasia and is characterized by severe impairments in all language modalities. The common specific injury sites include Broca's and Wernicke's areas, which can arise after massive strokes (3–5). Although some patients with global aphasia do not have cognitive deficits (6), they cannot express their thoughts correctly and appropriately, and their comprehension is severely restricted. The pattern of language impairment in patients with global aphasia is still controversial. For instance, Sarno proposed that patients with global aphasia retain no propositional language abilities (7). However, the measurements that they used emphasized too much on expressive language production and language comprehension to accurately reflect the language function of patients with severe aphasia (8). Several studies utilizing computerized visual communication showed that patients with global aphasia retain a degree of language ability (9–11). To date, there are still difficulties in assessing the residual language ability of patients with global aphasia.

The promotion of functional neuroimaging has led to a deeper understanding of patients with global aphasia. Functional magnetic resonance imaging (fMRI) is the most common imaging approach, which has helped reveal the damage pattern of brain networks and the mechanism underlying treatment-induced plasticity. For instance, researchers have found that the most consistently active regions during semantic word processing in patients with global aphasia are the left posterior temporal and right posterior parietal cortex. The recovery of the lexical-semantic system was associated predominantly with the activations of these regions (12). Resting-state fMRI (rs-fMRI) showed that patients with global aphasia had decreased interhemispheric and intra-hemispheric connectivity and enhanced connection between language network and cerebellar structures (13). These studies show that the utilization of neuroimaging techniques is feasible for research on global aphasia. Nevertheless, certain disadvantages of fMRI may limit its application in post-stroke aphasia (14). For example, motion artifacts will interfere with the neuron activation signals. In addition, the loud noise during scanning can affect language processing. Individuals with electronic medical implants are unsuited for an fMRI study. All of these factors affect the generalizability of the study.

Despite functional near-infrared spectroscopy (fNIRS) being a mature and feasible neuroimaging method, it is only now becoming an increasingly popular technique. Within the activated cortical region, oxyhemoglobin (HbO) increases and deoxyhemoglobin (HbR) decreases, and the changes in HbO and HbR can be measured by NIR light to reflect cortical activation. Two irreplaceable advantages of fNIRS for language studies are ecological validity and compatibility with other techniques (15). fNIRS can be compatible with facial and jaw movements for broader application in language production processes (16, 17). Moreover, it does not produce a magnetic field and is silent and comfortable for the user. Despite the feasibility and advantage, its application in aphasia research is restricted, especially for patients with global aphasia. In reality, for the past 20 years, although the number of published articles related to fNIRS has doubled every 3.5 years, only five of them have included individuals with aphasia. Moreover, more focus is placed on altered language functional patterns in patients with mild or moderate aphasia, and individuals with severe or global aphasia have not been included in prior studies to date. Systematic reviews also call for more articles to be published in areas related to aphasia and fNIRS (15).

In conclusion, fNIRS has the potential to significantly advance the field of rehabilitation for global aphasia. However, before it can be used as a therapeutic intervention, it is crucial to understand the activation patterns captured by fNIRS that are exhibited by patients with global aphasia. Therefore, this study applied fNIRS to capture different cortical activation in patients with global aphasia and healthy controls during the process of naming and repetition. We hypothesized that patients with global aphasia would show distinct cortical activation patterns from healthy controls. More specifically, we expected patients with global aphasia to show increased activation in the left hemisphere and decreased activation in other areas of the cortex relative to healthy controls. Therefore, in the present study, fNIRS was utilized to explore the differential cortical activation patterns between patients with global aphasia and healthy controls during the process of naming and repetition, which may provide a novel perspective on the hemodynamic response during language processing in these patients.

## Materials and methods

### Participants

A total of 14 older healthy individuals with a mean age of 57.61 years (SD: 8.66, age range: 49–77 years) and nine individuals with chronic left-hemisphere stroke-induced global aphasia (mean age; 58 ± 8.15 years, age range: 48–76 years; mean duration post onset: 18.67 ± 6.38 months, range: 9–30 months) were recruited from greater Shanghai area from January 2020 to September 2021. Handedness was assessed with the Edinburgh Inventory (18). All included subjects were native Mandarin speakers. The clinical characteristics between the two groups showed no difference (except for C-WAB). Supplementary Table 1 provides additional details on the clinical characteristics of each participant.

Participants in the stroke group were diagnosed with global aphasia based on the Western Aphasia Battery (Simplified Chinese version) (WAB-AQ; mean: 13.34 ± 6.92, range: 2.6–21.7), and the Non-Language-based Cognitive Assessment (NLCA) was used to exclude patients who had cognitive impairment (19) (mean: 74.11 ± 2.92, range: 70–79). Table 1 presents detailed demographic and clinical information of patients in the global aphasia group. All participants provided written informed consent, and the study was approved by the ethics committee of Huashan Hospital [CHiCTR2000038808]. Supplementary Table 2 provides additional details on WAB scores and lesions for each patient.

**Table 1:** Demographics for individuals with global aphasia.

**Patient**	**Age (years)**	**Sex**	**Dominant hand**	**Time since stroke (months)**	**Stroke type**	**C-WAB**	**NLCA**
PA1	52	Male	Right	22	Infarction	6.3	73
PA2	55	Male	Right	18	Infarction	4.7	70
PA3	48	Male	Right	12	Infarction	21.7	71
PA4	68	Male	Right	30	Hemorrhage	12.7	76
PA5	55	Male	Right	9	Hemorrhage	13	73
PA6	56	Female	Right	14	Infarction	19.8	75
PA7	54	Male	Right	21	Infarction	19.3	72
PA8	58	Male	Right	26	Hemorrhage	2.6	79
PA9	76	Female	Right	16	Hemorrhage	20	78

### Experimental procedure

**Figure 1:**
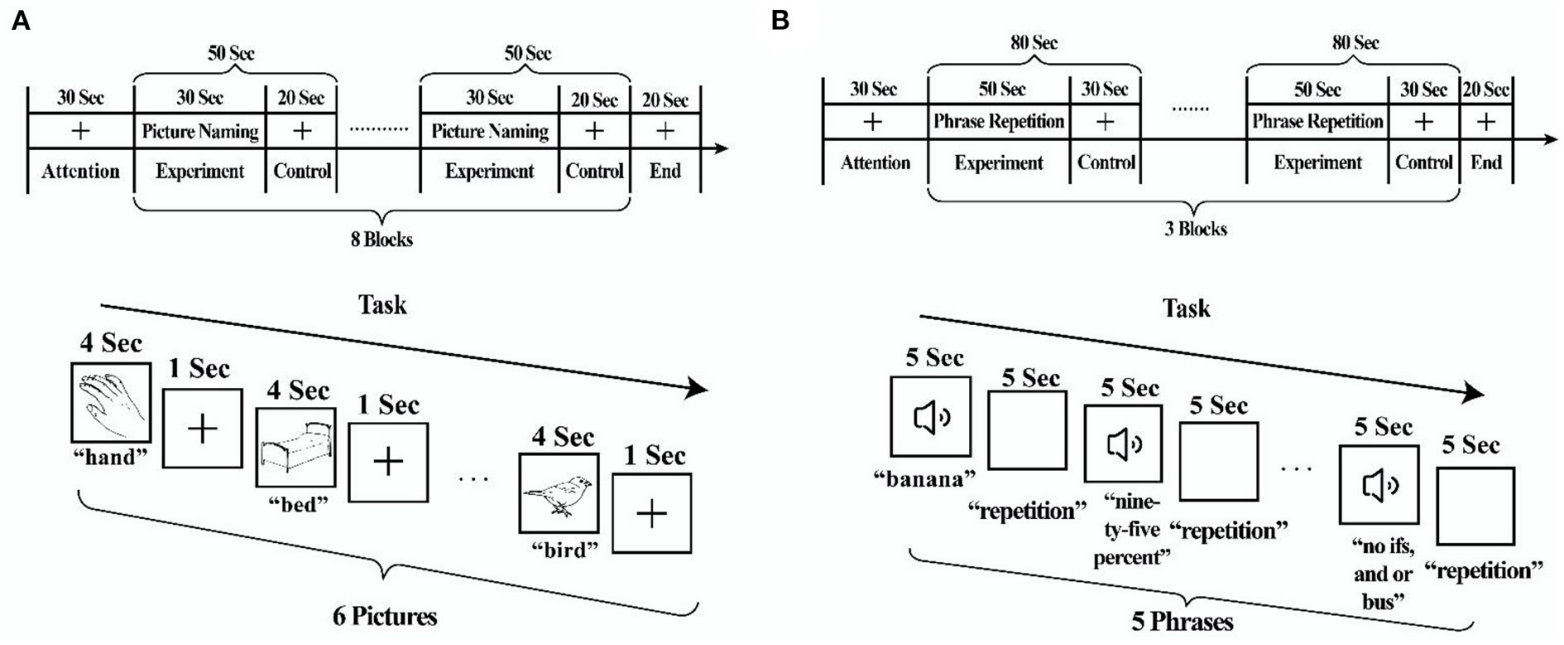
fNIRS behavioral tasks. **(A)** Picture-naming task. **(B)** Phrase repetition task.

During fNIRS scanning, participants completed two runs of picture-naming and phrase repetition tasks in consecutive order (Figure 1). In a practice session before the fNIRS scanning session, tasks were performed to familiarize the participants.

The picture-naming task (20) comprised 48 pictures from the S&V database of black and white line drawings (21). The task paradigm was a periodic block design with the experimental block (30 s) and control block (20 s). Each block was repeated eight times for an overall task time of 7.5–8 min/run. For the experimental blocks, participants were shown six pictures (e.g., hand) and asked to name them aloud. For the control blocks, participants were shown a black fixation crosshair. Responses during the picture-naming task were recorded to ensure accuracy.

The phrase repetition task (22) comprised 15 phrases selected from the Western Aphasia Battery or Boston Diagnostic Aphasia Examination. The task paradigm was a periodic block design with the experimental block (50 s) and control block (30 s). Each block was repeated three times for an overall task time of 5 min/run. For the experimental blocks, participants were asked to listen to five series of words carefully through earphones (e.g., banana) and subsequently had to repeat the phrases out aloud. For the control blocks, they were shown a black fixation crosshair. Supplementary Table 3 provides additional details on behavioral data for each participant.

In this experiment, continuous-wave fNIRS measurements (NirScan, Huichuang, China) were utilized to capture the HbO signals and HbR signals of the participants' scalp and cortex. The sampling frequency was 11 Hz, and the wavelengths were 730 and 850 nm.

**Figure 2:**
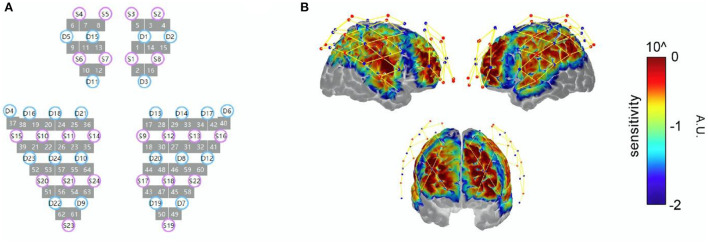
**(A)** fNIRS probe design; sources are represented by S, while detectors are represented by D and channels with lines between S and D. The arrangement of the optodes with an interoptode distance of 30 mm. **(B)** Sensitivity profile; sources are displayed with red dots, while detectors are displayed with blue dots and channels with yellow lines. Results of the Monte Carlo simulation based on 1 × 10^−8^ photons over the frontal, parietal, temporal, and occipital lobes: colors represent the spatial sensitivity of the fNIRS measurements. Warmer colors suggest higher sensitivity to the cortex, while cooler colors suggest lower sensitivity to the cortex.

The 64-channel probe (24 sources and 24 detectors) covered the frontal, temporal, parietal, and occipital lobes bilaterally. To obtain the Montreal Neurological Institute (MNI) coordinates of each fNIRS channel (represented by the midpoint location of the source–detector pair), the spatial coordinates of the sources, detectors, and anchor points (Cz, Nz, Iz, AL, and AR) were measured using the electromagnetic 3D digitizer system (FASTRAK, Polhemus, USA). Then, the cortical sensitivity of each channel was displayed on the MNI “Colin27” brain template by using the AtlasViewer (23) (Figure 2). Supplementary Table 4 shows the exact location of each channel.

Channels were assigned to the following regions of interest (ROIs) using their average MNI coordinate location: the dorsolateral prefrontal cortex (DLPFC), Broca's area (Broca), pre-motor and supplementary motor cortex (SMA), middle temporal gyrus (MTG), superior temporal gyrus (STG), supramarginal gyrus (SMG), and angular gyrus (AG). Table 2 presents information on the channel assignment to ROI.

**Table 2:** Channels assigned to regions of interest.

**ROI**	**Left hemisphere**	**Right hemisphere**
Dorsolateral prefrontal cortex (DLPFC)	6, 10, 11, 12	2, 4, 14, 16
Broca's area (Broca)	9	15
Pre-motor and supplementary motor cortex (SMA)	24, 25, 36	17, 18, 29
Middle temporal gyrus (MTG)	37	40
Superior temporal gyrus (STG)	38, 39	41, 42
Supramarginal gyrus (SMG)	52, 53, 55, 57	31, 46, 48, 59, 60
Angular gyrus (AG)	51	45, 58

### fNIRS data analysis and statistics

All fNIRS data were analyzed using HomER2 (24), NIRS-SPM (25), and BrainNet View (26). The processing stream included converting raw data to an optical density, and a 0.01–0.2 Hz Butterworth bandpass filter was applied to remove noise. The optical density was converted to HbO and HbR by using the modified Beer–Lambert law with a differential pathlength factor of 6. In this study, we used the HbO signal to denote the results, which generally has a better signal-to-noise ratio than the HbR signal (27). To estimate the task-related cortical activation, the HbO signals of each channel were analyzed using the general linear model (GLM) with regression to a hemodynamic response function (HRF) model with time and dispersion derivatives as modified for fNIRS. Beta-values served as contrasts, which were speculated using two-sample *t*-tests. A *p*-value of < 0.05 was considered to indicate significant differences between the HbO concentration in the experimental and control condition, but significant findings at *p* < 0.05 level that did not survive Bonferroni adjustment were interpreted and discussed given the exploratory nature of this pilot study. Moreover, to investigate the relationship between the accuracy of picture naming and cortical activation, we applied Spearman's correlation by using the latency of HbO concentration and the accuracy of the naming score. Data were analyzed using SPSS software version 26.0 (IBM Corporation, Armonk, NY, USA).

## Results

### Picture naming

**Figure 3:**
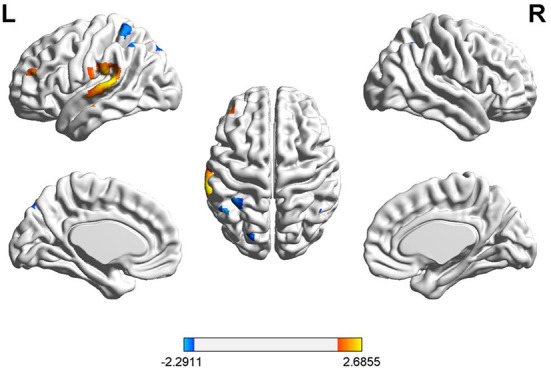
Cortical activation associated with the picture-naming task. HbO activation maps between patients with global aphasia and healthy individuals. The color bar represents the *t*-statistic. Warmer colors with positive values represent increases in HbO in patients with global aphasia compared with healthy individuals, while cooler colors with negative values represent opposite results (*p* < 0.05).

During the picture-naming task, patients with global aphasia showed significantly increased activation in channels 55 and 57, while significantly decreased activation was detected in channels 9, 37, and 39 in the left hemisphere. No channels in the right hemisphere showed significant differences between the two groups. Significant activations principally included classical left-hemisphere language areas. These were found in the left Broca's area, MTG, STG, and SMG. The details are shown in Figure 3 and Table 3.

**Table 3:** Exact location and intensity of the activation during the picture-naming task.

**Channel**	**MNI coordinates (X/Y/Z)**	**Cortical region**	**BA**	**Proportion**	**T-value**	***p*-value**
Ch.9	−47/47/18	L Broca	45	56.39%	2.37	0.027
Ch.37	−69/−13/−7	L MTG	21	85.44%	2.30	0.032
Ch.39	−69/−34/17	L STG	22	74.15%	2.69	0.014
Ch.55	−40/−50/67	L SMG	40	40.08%	−2.16	0.043
Ch.57	−50/−52/57	L SMG	40	89.84%	−2.29	0.032

**Figure 4:**
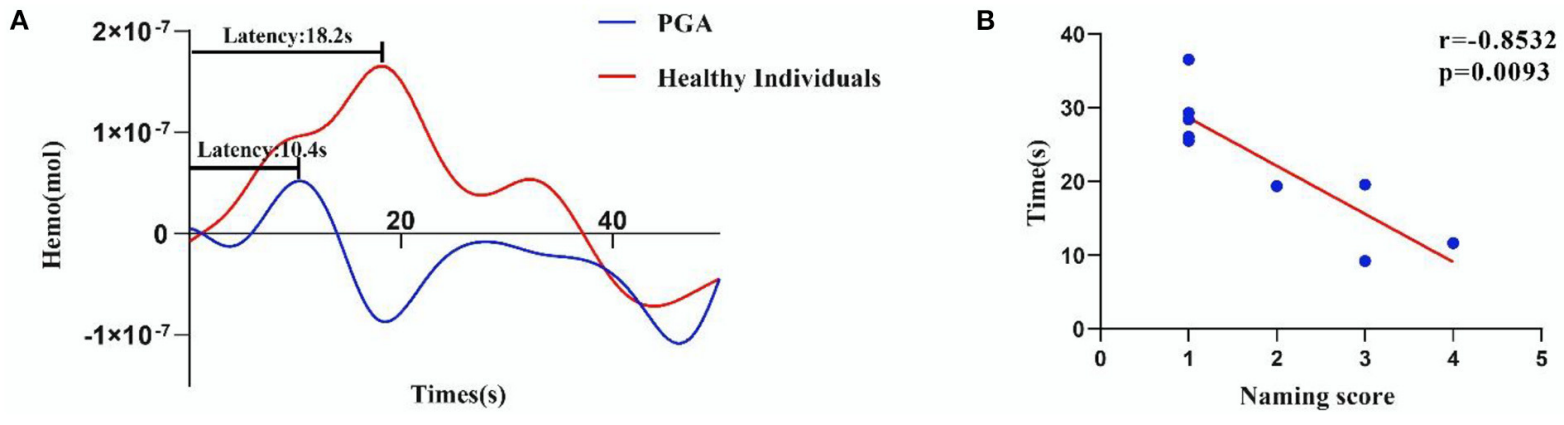
Significant correlation between the naming score and latency of HbO concentration in the left SMG (r = −0.8532, *p* = 0.0093). **(A)** The average HRF of the left SMG in patients with global aphasia and healthy individuals. **(B)** The accuracy of HbO concentration in the left SMG was negatively correlated with the naming score (*p* < 0.01) (The latency of HbO concentration and the naming score in some patients are very similar (refer to Supplementary Table 5 for more detail).

At the same time, we found that the latency of HbO concentration in the left SMG had a significant negative correlation with the naming score in patients with global aphasia (r = −0.8532, *p* = 0.0093) (Figure 4).

### Phrase repetition

**Figure 5:**
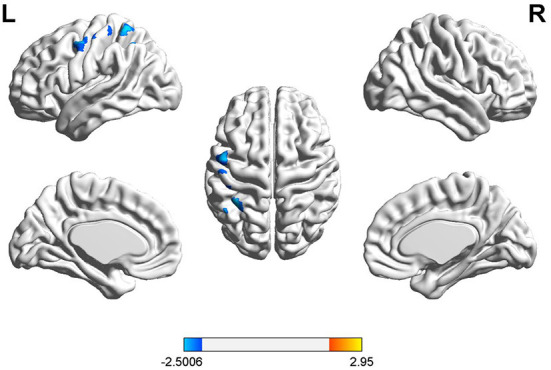
Cortical activation associated with phrase repetition task. HbO activation maps between patients with global aphasia and healthy individuals, and the color bar represents the *t*-statistic. Cooler colors with negative values represent decreases in HbO in patients with global aphasia compared with healthy individuals (*p* < 0.05).

During the phrase repetition task, a significant HbO decrease was found in three channels (25, 55, and 57) in the left hemisphere of patients with global aphasia relative to healthy individuals. These were detected in the left pre-motor, SMA, and SMG. Anatomical labeling and spatial probability were estimated for the significantly activated channels (Figure 5 and Table 4).

**Table 4:** Exact location and intensity of activation during phrase repetition task.

**Channel**	**MNI coordinates (X/Y/Z)**	**Cortical region**	**BA**	**Proportion**	**T-value**	***p*-value**
Ch.25	−50/−1/55	L SMA	45	98.39%	−2.43	0.024
Ch.55	−40/−50/67	L SMG	40	40.08%	−2.50	0.021
Ch.57	−50/−52/57	L SMG	40	89.84%	−2.39	0.026

## Discussion

In the present study, we used fNIRS to assess and compare the different cortical activation patterns for picture naming and phrase repetition between patients with global aphasia and healthy controls. In the naming task, patients with global aphasia demonstrated increased activation in the left Broca's area, MTG, and STG, but a decreased activation in the left SMG relative to controls. Furthermore, the latency of the HbO hemodynamic curve in the SMG region was highly negatively correlated with the naming score of patients with global aphasia. On the contrary, the SMA and SMG in the left hemisphere exhibited a decrease in cortical activation during the phrase repetition task in patients with global aphasia compared with healthy controls. Notably, we found that the left SMG may play a critical role in both tasks for patients with global aphasia.

### Cortical activation patterns for language captured by fNIRS

Our results suggest that the left Broca's area, MTG, and STG play key roles in picture naming, which is consistent with previous fNIRS studies on healthy controls (28–30), while at the same time, a retrospective multicenter study reported one cluster of anomia in the posterior STG and MTG, and the other within the Broca's area (31). Meanwhile, the results from an earlier fNIRS study revealed that the prefrontal cortex was more active during naming in patients with post-stroke aphasia than in healthy controls and those with non-aphasia (32). These results supported our finding that the left prefrontal lobe (Broca's area) is more activated during naming tasks in patients with global aphasia, while our present study showed that the over-activation was also detected in the Wernicke's area. We believe that the over-activation was attributed to the overcompensation in the phonological and semantic processes in the patients with global aphasia (33).

In this study, patients with global aphasia showed decreased activation of left SMA relative to controls when repeating automatic phrases vs. looking at a fixation cross. These results are novel because very few fNIRS studies have investigated cortical activation during automatic phrase repetition in people with aphasia (34). Our study isolated the location of brain activation during repetition in patients with global aphasia. These preliminary findings are reasonable in the context of prior fNIRS studies showing the role of the SMA in the recovery of thalamic aphasia (35). Some even suggest that the SMA is important for language recovery in aphasia given its connection to the left inferior frontal gyrus, or Broca's area through the arcuate fasciculus, a key tract for language processing (36). In conclusion, we believe that the left SMA plays a key role not only in mild or moderate aphasia but also in a global aphasia.

### Left SMG plays a fundamental role in picture naming and phrase repetition

In both naming and phrase repetition tasks, we found a significant decrease in the deactivation of the left SMG. This may be related to the different degrees of the loss of “repetition ability” and “naming ability” in patients with global aphasia. This loss of language function may be similar to the phenomenon of “anomia” or “conduction aphasia” induced by direct cortical electrical stimulation (DES), which is the gold standard for defining a patient's speech cortex (37). During the picture-naming task, DES on the left SMG resulted in phonological or semantic discontinuation (38). Studies have also found that DES on the left SMG leads to conduction aphasia (39). Some researchers have found that restraining the left SMG by repetitive transcranial magnetic stimulation (rTMS) will reduce language performance (40).

Moreover, we found the latency of the left SMG was negatively correlated with the ability to name, similar to the results from a previous fMRI study (41). In a recent fNIRS study performed by Gilmore et al., the left SMG was also reported to be involved in semantic features and picture-naming tasks (42), while differences should be noted between the two studies. Gilmore et al. enrolled patients with an average AQ of 67.17 (42.20–93.20), while in our present study, patients with an average AQ of 13.34 (2.6–21.7) were included. The fact that in aphasia patients with various severity, the left SMG has been constantly found to be activated in language tasks, stressing its vital role in language function. More importantly, the left SMG activation may be occurring because it is likely the primary area of the spared cortex in individuals with global aphasia with large left hemisphere damage or not actually be a signal from the cortex (may be the scalp signal over lesion). However, this cannot be confirmed in this study because MRI was not obtained. The results of the alternate analysis (excluding the area of the frank lesion from analysis) are reported in Supplementary Tables 6, 7 and Figures 1, 2. To conclude, the left SMG was suggested to represent a general cognitive region required for language processing in various aphasic situations.

Studies have started using rTMS to stimulate either the dominant or non-dominant hemisphere in aphasia to support language recovery (43–47). The stimulation site of rTMS in patients with aphasia can be selected by using the results of fNIRS, as also reported by Chang et al. (48). Our findings are congruent with a study conducted by Ren, wherein patients with global aphasia were treated with low-frequency rTMS at the temporoparietal region (CP6), which includes the posterior STG (pSTG) and SMG in the right hemisphere. They reported an improvement in the WAB-AQ score, spontaneous speech, and repetition ability when compared to the sham groups (49). This may indicate that SMG is a potential target in noninvasive brain stimulation for the treatment of patients with global aphasia, based on the fNIRS mapping method.

## Conclusion

The current study utilized fNIRS to assess brain reorganization in patients with global aphasia. We believe that the decline of language ability in patients with global aphasia may be related to the abnormal decline of the left SMG, which may play a key role in language, especially in naming and repetition ability. Our results indicate the specific brain functional pattern in patients with global aphasia, especially in naming and repetition and in facilitating the wider application of fNIRS. Moreover, these findings may be applied to better assist patients with global aphasia and are expected to contribute to the development of noninvasive brain stimulation for the treatment of such patients.

## Limitations

This study has some limitations. A disadvantage of fNIRS that is not unique to this study is that it measures signals ~1.5-cm deep into the cortex, meaning that the contribution of subcortical structures in naming tasks in global aphasia could not be captured in this study. Nevertheless, the cortical coverage in this study was greater than in other aphasia studies to date [e.g., 64 channels in this study vs. 56 channels in Gilmore et al. (42)]. Similarly, methods for managing the lesion in fNIRS studies in aphasia are still emerging. In this study, we did not conduct a structural MRI and could not map the location of participants' lesions. Thus, we were unable to confirm whether we were obtaining spurious signals from the scalp vs. the cortex when the channels were measuring an area of the frank lesion. Beyond that, our probe did not include short-separation channels, meaning that we were unable to regress out physiological noise from the hemodynamic response, while this study used Wavelet-MDL and generalized linear model (GLM)-based analysis, which can regress out some physiological noise and manage delayed activation at other regions. In addition, the results of the alternate analysis [average all of the long-separation channels together and divide all the channels by that signal to adjust for physiological noise (50, 51)] were in line with what was found in the primary analyses reported in the article; we get a similar cortical activation pattern (see Supplementary Table 8 and Figures 3, 4), and the result further confirms our scientific hypothesis that the left SMG plays a key regulatory role in the process of naming and repetition of patients with global aphasia. The use of short-separation regression channels will be an important step in the future to increase the precision of our findings. Furthermore, we were unable to carry out structural MRI, so we could not measure the scalp-to-cortex distance (i.e., greater distance, less likely capturing the signal from the cortex), which increases with age and brain injury—another factor impacting the validity of our findings that must be taken into consideration when interpreting the results. Another limitation worth discussing is that the activation we captured when contrasting picture naming with a fixation cross (silent task) could reflect engagement for speech production vs. language processing. A tighter contrast of contrasting picture naming with a control condition that also involves speech production but no naming (e.g., repeating their name or a word they can reliably say) would provide information about language processing/lexical retrieval in people with aphasia. More ecologically valid language tasks (e.g., covert word retrieval to semantic cues) can also be used in future studies to improve the measurement of various linguistic processes important for word retrieval. Despite these challenges, the results from this research pave the way for future work investigating patients with global aphasia by fNIRS, using high-density probes, and a larger sample size, together with more complex and diverse language tasks.

## Data availability statement

The raw data supporting the conclusions of this article will be made available by the authors, without undue reservation.

## Ethics statement

The studies involving human participants were reviewed and approved by Huashan Hospital Institutional Review Board. The patients/participants provided their written informed consent to participate in this study.

## Author contributions

HL and JL performed the experiments, analyzed the fNIRS data, conducted all statistical analyses, and wrote all sections of the manuscript. ST, SF, and TW helped to revise the manuscript, figures, and tables. HQ and GL advised on the statistical analyses. YZ helped conceive the illustrations and revised the main body of the manuscript. RH and YW served as the primary scientific mentors to HL and were involved in experiment conceptualization, experimental procedure design, and manuscript review. All authors have declared that no competing interests exist and approved the submitted version.
