# Supplementary material for: Language reorganization patterns in global aphasia–evidence from fNIRS
Source: Front Neurol. 2023 Jan 6;13:1025384. doi: 10.3389/fneur.2022.1025384 (PMC9853054; doi:10.3389/fneur.2022.1025384)
Supplement: Supplementary file 1 [file Table_1.DOCX]

# Supplementary Table 1. Clinical characteristics for each participant

| **Participant** | **Age(years)** | **Gender** | **Dominant hand** | **C-WAB** | **NLCA** |
| --- | --- | --- | --- | --- | --- |
| **PA1** | **52** | **Male** | **Right** | **6.3** | **73** |
| **PA2** | **55** | **Male** | **Right** | **4.7** | **70** |
| **PA3** | **48** | **Male** | **Right** | **21.7** | **71** |
| **PA4** | **68** | **Male** | **Right** | **12.7** | **76** |
| **PA5** | **55** | **Male** | **Right** | **13** | **73** |
| **PA6** | **56** | **Female** | **Right** | **19.8** | **75** |
| **PA7** | **54** | **Male** | **Right** | **19.3** | **72** |
| **PA8** | **58** | **Male** | **Right** | **2.6** | **79** |
| **PA9** | **76** | **Female** | **Right** | **20** | **78** |
| **HC1** | **77** | **Female** | **Right** | **100** | **77** |
| **HC2** | **52** | **Male** | **Right** | **100** | **72** |
| **HC3** | **52** | **Male** | **Right** | **100** | **74** |
| **HC4** | **66** | **Male** | **Right** | **99.7** | **77** |
| **HC5** | **62** | **Male** | **Right** | **100** | **71** |
| **HC6** | **49** | **Male** | **Right** | **97.1** | **76** |
| **HC7** | **62** | **Male** | **Right** | **96** | **74** |
| **HC8** | **58** | **Male** | **Right** | **98** | **79** |
| **HC9** | **51** | **Male** | **Right** | **99.4** | **70** |
| **HC10** | **66** | **Male** | **Right** | **100** | **80** |
| **HC11** | **49** | **Female** | **Right** | **100** | **72** |
| **HC12** | **45** | **Female** | **Right** | **100** | **73** |
| **HC13** | **60** | **Male** | **Right** | **97.2** | **79** |
| **HC14** | **58** | **Male** | **Right** | **98** | **80** |

*Note.* PA = patient, HC = Healthy Control
