# Supplementary material for: Language reorganization patterns in global aphasia–evidence from fNIRS
Source: Front Neurol. 2023 Jan 6;13:1025384. doi: 10.3389/fneur.2022.1025384 (PMC9853054; doi:10.3389/fneur.2022.1025384)
Supplement: Supplementary file 2 [file Table_2.DOCX]

# Supplementary Table 2. WAB scores and lesions of each patient

| **Patient** | **Fluency**  **+ Information** | **Aud.Comprehension** | **Repetition** | **Naming** | **AQ**  **(Aphasia Quotient)** | **Type of Aphasia** | **Lesion** |
| --- | --- | --- | --- | --- | --- | --- | --- |
| **PA1** | **1+1** | **0.65** | **0.2** | **0.3** | **6.3** | **Global** | **Left frontal and temporal lobes** |
| **PA2** | **2+0** | **0.15** | **0.1** | **0.1** | **4.7** | **Global** | **Left parietal and temporal lobes** |
| **PA3** | **4+2** | **1.25** | **1.8** | **1.8** | **21.7** | **Global** | **Left parietal and temporal lobes** |
| **PA4** | **2+1** | **0.75** | **0.2** | **2.4** | **12.7** | **Global** | **Left middle cerebral artery region** |
| **PA5** | **2+1** | **0** | **1.8** | **1.7** | **13** | **Global** | **Left basal ganglia** |
| **PA6** | **4+2** | **1.5** | **2** | **0.4** | **19.8** | **Global** | **Left frontal lobe** |
| **PA7** | **4+2** | **1.85** | **1.2** | **0.6** | **19.3** | **Global** | **Left frontal and parietal lobes** |
| **PA8** | **1+0** | **0** | **0** | **0.3** | **2.6** | **Global** | **Left basal ganglia and corona radiata** |
| **PA9** | **4+2** | **0** | **2** | **2** | **20** | **Global** | **Left temporal lobe** |
