# Supplementary material for: Language reorganization patterns in global aphasia–evidence from fNIRS
Source: Front Neurol. 2023 Jan 6;13:1025384. doi: 10.3389/fneur.2022.1025384 (PMC9853054; doi:10.3389/fneur.2022.1025384)
Supplement: Supplementary file 3 [file Table_3.DOCX]

# Supplementary Table 3. Behavioral Data of each participant

| **Participant** | **Accuracy of naming** | **Accuracy of repetition** |
| --- | --- | --- |
| **PA1** | **1/48** | **1/15** |
| **PA2** | **1/48** | **1/15** |
| **PA3** | **3/48** | **1/15** |
| **PA4** | **3/48** | **2/15** |
| **PA5** | **2/48** | **3/15** |
| **PA6** | **1/48** | **2/15** |
| **PA7** | **1/48** | **1/15** |
| **PA8** | **1/48** | **0/15** |
| **PA9** | **4/48** | **4/15** |
| **HC1** | **48/48** | **15/15** |
| **HC2** | **48/48** | **15/15** |
| **HC3** | **48/48** | **15/15** |
| **HC4** | **48/48** | **15/15** |
| **HC5** | **48/48** | **15/15** |
| **HC6** | **48/48** | **15/15** |
| **HC7** | **48/48** | **15/15** |
| **HC8** | **48/48** | **15/15** |
| **HC9** | **48/48** | **15/15** |
| **HC10** | **48/48** | **15/15** |
| **HC11** | **48/48** | **15/15** |
| **HC12** | **48/48** | **15/15** |
| **HC13** | **48/48** | **15/15** |
| **HC14** | **48/48** | **15/15** |

*Note.* PA = patient, HC = Healthy Control
