# Supplementary material for: Language reorganization patterns in global aphasia–evidence from fNIRS
Source: Front Neurol. 2023 Jan 6;13:1025384. doi: 10.3389/fneur.2022.1025384 (PMC9853054; doi:10.3389/fneur.2022.1025384)
Supplement: Supplementary file 4 [file Table_4.DOCX]

# Supplementary Table 4. The exact location for each channel

| **Channel** | **MNI coordinates**  **(X/Y/Z)** | **Cortical region** | **BA** | **Proportion** |
| --- | --- | --- | --- | --- |
| **Ch.1** | **20/67/25** | **R Frontopolar area** | **10** | **94.90%** |
| **Ch.2** | **15/51/48** | **R DLPFC** | **9** | **100.00%** |
| **Ch.3** | **41/63/-5** | **R Frontopolar area** | **10** | **42.86%** |
| **Ch.4** | **50/52/-5** | **R DLPFC** | **46** | **89.14%** |
| **Ch.5** | **24/70/-7** | **R Orbitofrontal area** | **11** | **92.05%** |
| **Ch.6** | **-47/52/-4** | **L DLPFC** | **46** | **89.40%** |
| **Ch.7** | **-38/63/-4** | **L Frontopolar area** | **10** | **52.69%** |
| **Ch.8** | **-24/69/-4** | **L Orbitofrontal area** | **11** | **79.07%** |
| **Ch.9** | **-47/47/18** | **L Broca's area** | **45** | **56.39%** |
| **Ch.10** | **-35/43/40** | **L DLPFC** | **9** | **58.88%** |
| **Ch.11** | **-39/58/19** | **L DLPFC** | **46** | **81.71%** |
| **Ch.12** | **-22/49/43** | **L DLPFC** | **9** | **97.74%** |
| **Ch.13** | **-27/64/22** | **L Frontopolar area** | **10** | **64.09%** |
| **Ch.14** | **36/59/25** | **R DLPFC** | **46** | **83.61%** |
| **Ch.15** | **47/49/24** | **R Broca's area** | **45** | **51.65%** |
| **Ch.16** | **29/43/46** | **R DLPFC** | **9** | **100.00%** |
| **Ch.17** | **38/0/65** | **R SMA** | **6** | **95.80%** |
| **Ch.18** | **40/-23/71** | **R M1** | **4** | **71.48%** |
| **Ch.19** | **-68/-8/23** | **L Subcentral area** | **43** | **77.96%** |
| **Ch.20** | **-65/-6/34** | **L Subcentral area** | **43** | **65.10%** |
| **Ch.21** | **-68/-32/32** | **L S1** | **2** | **49.38%** |
| **Ch.22** | **-65/-30/44** | **L S1** | **2** | **51.88%** |
| **Ch.23** | **-51/-25/63** | **L S1** | **3** | **84.31%** |
| **Ch.24** | **-58/-3/46** | **L SMA** | **6** | **55.72%** |
| **Ch.25** | **-50/-1/55** | **L SMA** | **6** | **98.39%** |
| **Ch.26** | **-58/-27/53** | **L S1** | **3** | **79.54%** |
| **Ch.27** | **58/-28/56** | **R S1** | **1** | **80.39%** |
| **Ch.28** | **47/-1/60** | **R SMA** | **6** | **87.20%** |
| **Ch.29** | **57/-3/51** | **R SMA** | **6** | **78.18%** |
| **Ch.30** | **50/-25/65** | **R S1** | **3** | **69.53%** |
| **Ch.31** | **66/-30/48** | **R SMG** | **40** | **51.08%** |
| **Ch.32** | **70/-30/34** | **R S1** | **2** | **51.40%** |
| **Ch.33** | **65/-4/39** | **R Subcentral area** | **43** | **32.64%** |
| **Ch.34** | **69/-6/27** | **R Subcentral area** | **43** | **93.61%** |
| **Ch.35** | **-38/-23/71** | **L M1** | **4** | **73.31%** |
| **Ch.36** | **-40/1/63** | **L SMA** | **6** | **98.11%** |
| **Ch.37** | **-69/-13/-7** | **L MTG** | **21** | **85.44%** |
| **Ch.38** | **-67/-11/9** | **L STG** | **22** | **77.53%** |
| **Ch.39** | **-69/-34/17** | **L STG** | **22** | **74.15%** |
| **Ch.40** | **69/-5/-7** | **R MTG** | **21** | **90.13%** |
| **Ch.41** | **72/-31/15** | **R STG** | **22** | **86.85%** |
| **Ch.42** | **69/-7/8** | **R STG** | **22** | **60.13%** |
| **Ch.43** | **29/-62/70** | **R SAC** | **7** | **100.00%** |
| **Ch.44** | **35/-45/72** | **R S1** | **1** | **49.82%** |
| **Ch.45** | **46/-67/53** | **R AG** | **39** | **60.47%** |
| **Ch.46** | **54/-51/56** | **R SMG** | **40** | **94.62%** |
| **Ch.47** | **38/-64/62** | **R SAC** | **7** | **84.86%** |
| **Ch.48** | **44/-48/63** | **R SMG** | **40** | **59.22%** |
| **Ch.49** | **34/-81/46** | **R V3** | **19** | **51.87%** |
| **Ch.50** | **27/-79/55** | **R SAC** | **7** | **90.48%** |
| **Ch.51** | **-44/-75/46** | **L AG** | **39** | **60.26%** |
| **Ch.52** | **-62/-55/37** | **L SMG** | **40** | **52.76%** |
| **Ch.53** | **-58/-53/48** | **L SMG** | **40** | **80.61%** |
| **Ch.54** | **-27/-69/65** | **L SAC** | **7** | **100.00%** |
| **Ch.55** | **-40/-50/67** | **L SMG** | **40** | **40.80%** |
| **Ch.56** | **-35/-73/55** | **L SAC** | **7** | **89.43%** |
| **Ch.57** | **-50/-52/57** | **L SMG** | **40** | **89.84%** |
| **Ch.58** | **51/-69/45** | **R AG** | **39** | **96.92%** |
| **Ch.59** | **60/-51/49** | **R SMG** | **40** | **87.45%** |
| **Ch.60** | **64/-53/36** | **R SMG** | **40** | **54.42%** |
| **Ch.61** | **-16/-81/56** | **L SAC** | **7** | **87.70%** |
| **Ch.62** | **-22/-86/47** | **L V3** | **19** | **68.18%** |
| **Ch.63** | **-20/-66/70** | **L SAC** | **7** | **100.00%** |
| **Ch.64** | **-33/-46/72** | **L SAC** | **7** | **51.92%** |

***Note.* DLPFC =Dorsolateral prefrontal cortex, M1= Primary Motor Cortex, S1=Primary Somatosensory Cortex, SMG = supramarginal gyrus, MTG = middle temporal gyrus, STG= Superior Temporal Gyrus, AG = angular gyrus, SAC= Somatosensory Association Cortex.**
