# Supplementary material for: Language reorganization patterns in global aphasia–evidence from fNIRS
Source: Front Neurol. 2023 Jan 6;13:1025384. doi: 10.3389/fneur.2022.1025384 (PMC9853054; doi:10.3389/fneur.2022.1025384)
Supplement: Supplementary file 5 [file Table_5.DOCX]

# Supplementary Table 5. Naming score and latency of HbO concentration in left SMG of each patient

| **Patient** | **Naming score** | **Latency of HbO concentration in left SMG (s)** |
| --- | --- | --- |
| **PA1** | **1** | **29.31818** |
| **PA2** | **1** | **25.5** |
| **PA3** | **3** | **9.204545** |
| **PA4** | **3** | **19.59091** |
| **PA5** | **2** | **19.38636** |
| **PA6** | **1** | **26.09091** |
| **PA7** | **1** | **28.43182** |
| **PA8** | **1** | **36.54545** |
| **PA9** | **4** | **11.63636** |
