# Supplementary material for: Language reorganization patterns in global aphasia–evidence from fNIRS
Source: Front Neurol. 2023 Jan 6;13:1025384. doi: 10.3389/fneur.2022.1025384 (PMC9853054; doi:10.3389/fneur.2022.1025384)
Supplement: Supplementary file 6 [file Table_6.DOCX]

**Supplementary Table 6. Management of the lesion in each patient by turning channels off based on lobe location**

| **Patient** | **PA1** | **PA2** | **PA3** | **PA4** | **PA5** | **PA6** | **PA7** | **PA8** | **PA9** |
| --- | --- | --- | --- | --- | --- | --- | --- | --- | --- |
| **L DLPFC** | **Available** | **Available** | **Available** | **Available** | **Available** | **n/a** | **Available** | **Available** | **Available** |
| **R DLPFC** | **Available** | **Available** | **Available** | **Available** | **Available** | **Available** | **Available** | **Available** | **Available** |
| **L Broca** | **n/a** | **Available** | **Available** | **n/a** | **Available** | **n/a** | **n/a** | **Available** | **Available** |
| **R Broca** | **Available** | **Available** | **Available** | **Available** | **Available** | **Available** | **Available** | **Available** | **Available** |
| **L SMA** | **Available** | **Available** | **Available** | **n/a** | **Available** | **Available** | **Available** | **Available** | **Available** |
| **R SMA** | **Available** | **Available** | **Available** | **Available** | **Available** | **Available** | **Available** | **Available** | **Available** |
| **L MTG** | **n/a** | **n/a** | **n/a** | **n/a** | **Available** | **Available** | **Available** | **Available** | **n/a** |
| **R MTG** | **Available** | **Available** | **Available** | **Available** | **Available** | **Available** | **Available** | **Available** | **Available** |
| **L STG** | **n/a** | **n/a** | **n/a** | **n/a** | **Available** | **Available** | **Available** | **Available** | **n/a** |
| **R STG** | **Available** | **Available** | **Available** | **Available** | **Available** | **Available** | **Available** | **Available** | **Available** |
| **L SMG** | **Available** | **n/a** | **n/a** | **Available** | **Available** | **Available** | **n/a** | **Available** | **Available** |
| **R SMG** | **Available** | **Available** | **Available** | **Available** | **Available** | **Available** | **Available** | **Available** | **Available** |
| **L AG** | **Available** | **n/a** | **n/a** | **Available** | **Available** | **Available** | **n/a** | **Available** | **Available** |
| **R AG** | **Available** | **Available** | **Available** | **Available** | **Available** | **Available** | **Available** | **Available** | **Available** |

*Note.* PA = patient, N/A = Not available, L DLPFC = Left Dorsolateral prefrontal cortex, R DLPFC = Right Dorsolateral prefrontal cortex, L Broca = Left Broca's area, R Broca = Right Broca's area, L MTG = Left middle temporal gyrus, R MTG = Right middle temporal gyrus, L SMG = Left supramarginal gyrus, R SMG = Right supramarginal gyrus, L STG= Left Superior Temporal Gyrus, R STG= Right Superior Temporal Gyrus, L AG = Left angular gyrus, R AG = Right angular gyrus.
