# Supplementary material for: Language reorganization patterns in global aphasia–evidence from fNIRS
Source: Front Neurol. 2023 Jan 6;13:1025384. doi: 10.3389/fneur.2022.1025384 (PMC9853054; doi:10.3389/fneur.2022.1025384)
Supplement: Supplementary file 7 [file Table_7.DOCX]

**Supplementary Table 7. The exact location and intensity of the activation during tasks by turning channels off based on lobe location**

| Task | Channel | MNI coordinates  (X/Y/Z) | | Cortical  region | BA | Proportion | T-value |
| --- | --- | --- | --- | --- | --- | --- | --- |
| Naming | Ch.9 | -47/47/18 | L Broca | | 45 | 56.39% | 2.34 |
| Naming | Ch.37 | -69/-13/-7 | L MTG | | 21 | 85.44% | 2.35 |
| Naming | Ch.39 | -69/-34/17 | L STG | | 22 | 74.15% | 2.74 |
| Naming | Ch.53 | -58/-53/48 | L SMG | | 40 | 80.61% | -2.59 |
| Naming | Ch.55 | -40/-50/67 | L SMG | | 40 | 40.08% | -2.14 |
| Naming | Ch.57 | -50/-52/57 | L SMG | | 40 | 89.84% | -2.28 |
| Repetition | Ch.25 | -50/-1/55 | L SMA | | 45 | 98.39% | -2.22 |
| Repetition | Ch.55 | -40/-50/67 | L SMG | | 40 | 40.08% | -2.22 |
