# Supplementary material for: Language reorganization patterns in global aphasia–evidence from fNIRS
Source: Front Neurol. 2023 Jan 6;13:1025384. doi: 10.3389/fneur.2022.1025384 (PMC9853054; doi:10.3389/fneur.2022.1025384)
Supplement: Supplementary file 8 [file Table_8.DOCX]

**Supplementary Table 8. The exact location and the intensity of the activation during tasks by average all of the long-separation channels together and divide all the channels by that signal**

| Task | Channel | MNI coordinates  (X/Y/Z) | | Cortical  region | BA | Proportion | T-value |
| --- | --- | --- | --- | --- | --- | --- | --- |
| Naming | Ch.53 | -58/-53/48 | L SMG | | 40 | 80.61% | -2.63 |
| Repetition | Ch.25 | -50/-1/55 | L SMA | | 45 | 98.39% | -2.20 |
| Repetition | Ch.53 | -58/-53/48 | L SMG | | 40 | 80.61% | -2.25 |
| Repetition | Ch.55 | -40/-50/67 | L SMG | | 40 | 40.08% | -2.26 |
| Repetition | Ch.57 | -50/-52/57 | L SMG | | 40 | 89.84% | -2.44 |
