# Supplementary figures and images for: Language reorganization patterns in global aphasia–evidence from fNIRS
Source: Front Neurol. 2023 Jan 6;13:1025384. doi: 10.3389/fneur.2022.1025384 (PMC9853054; doi:10.3389/fneur.2022.1025384)

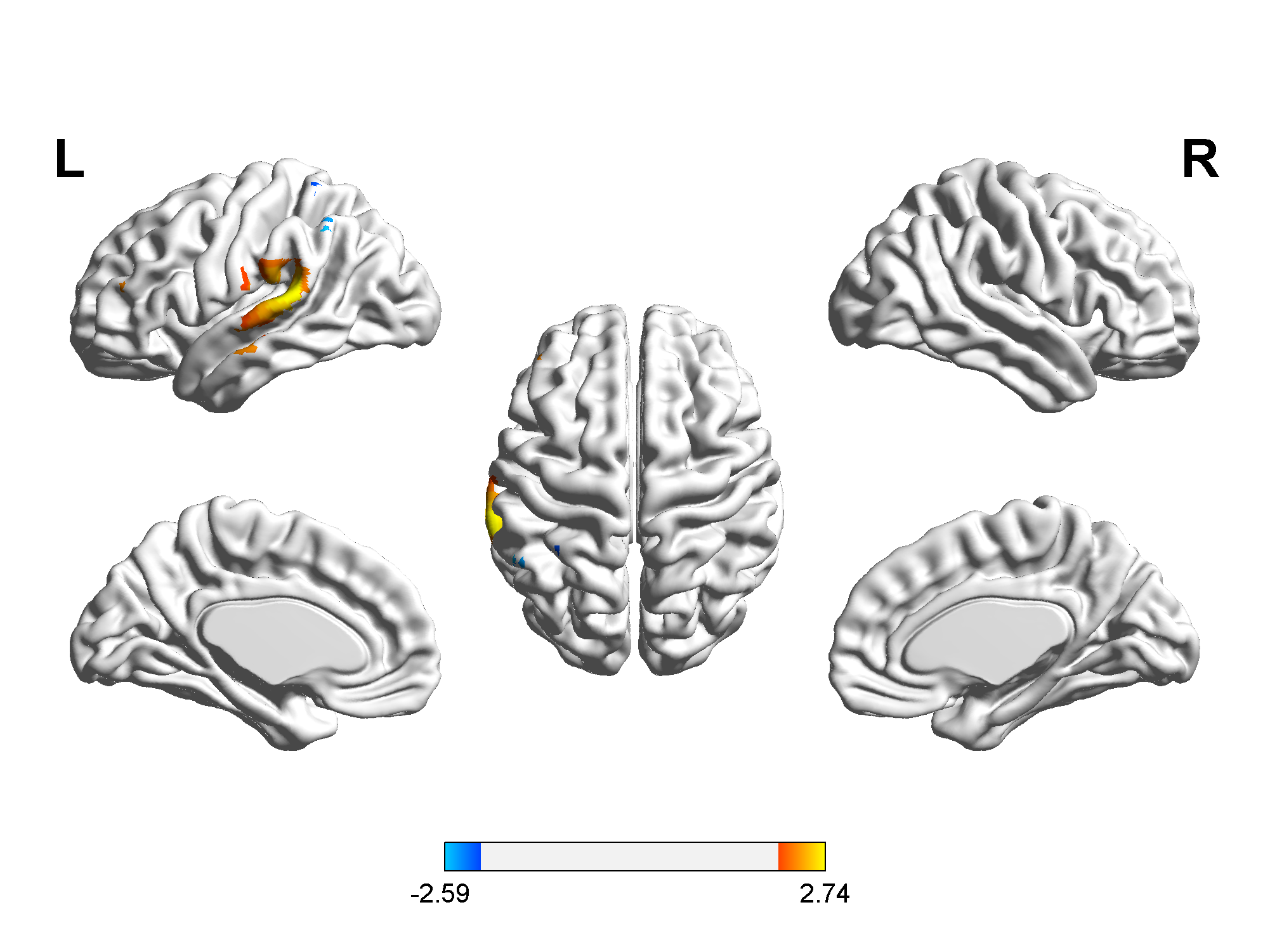

Supplement: Supplementary file 9 [file Image_1.TIF]

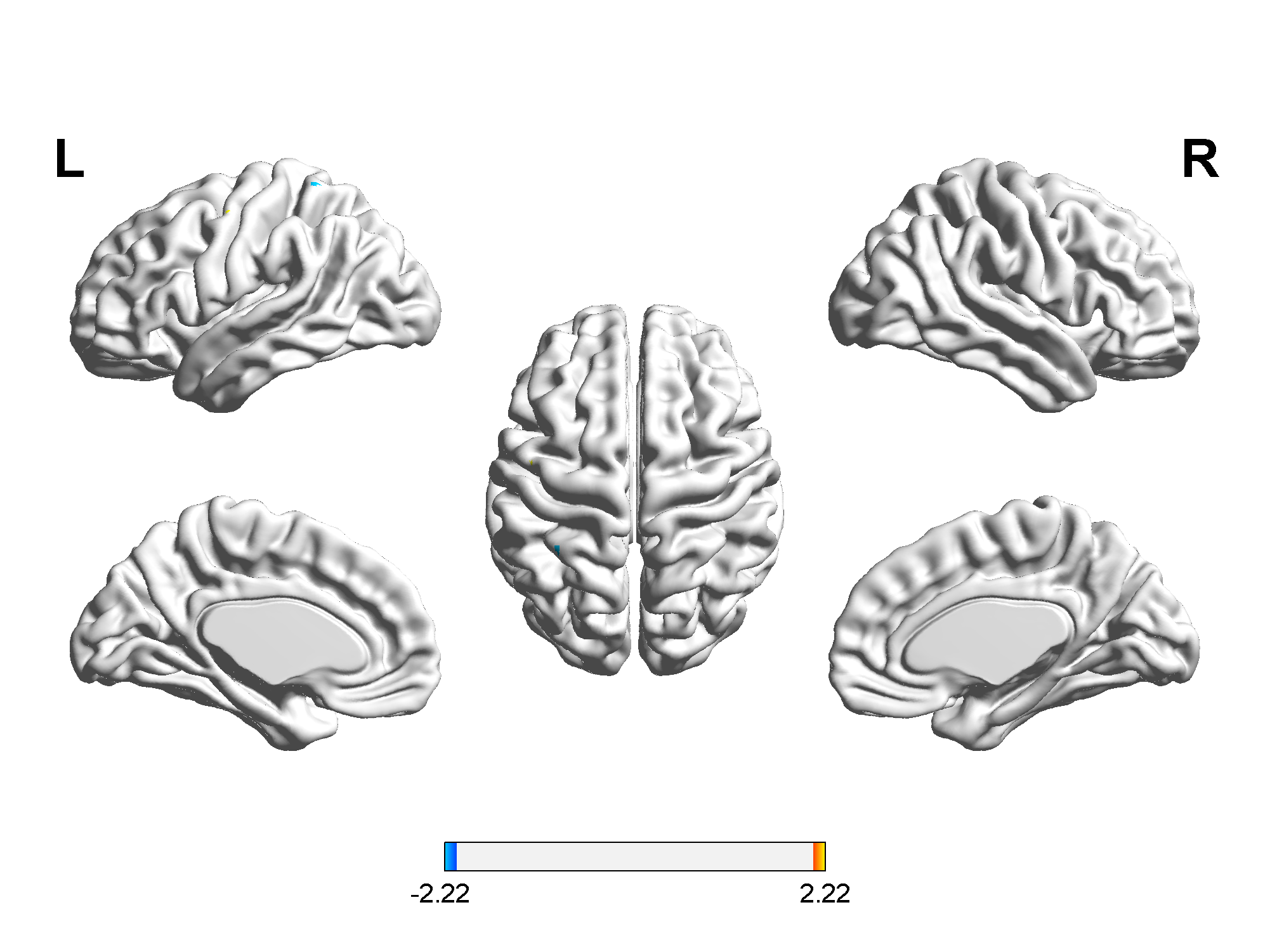

Supplement: Supplementary file 10 [file Image_2.TIF]

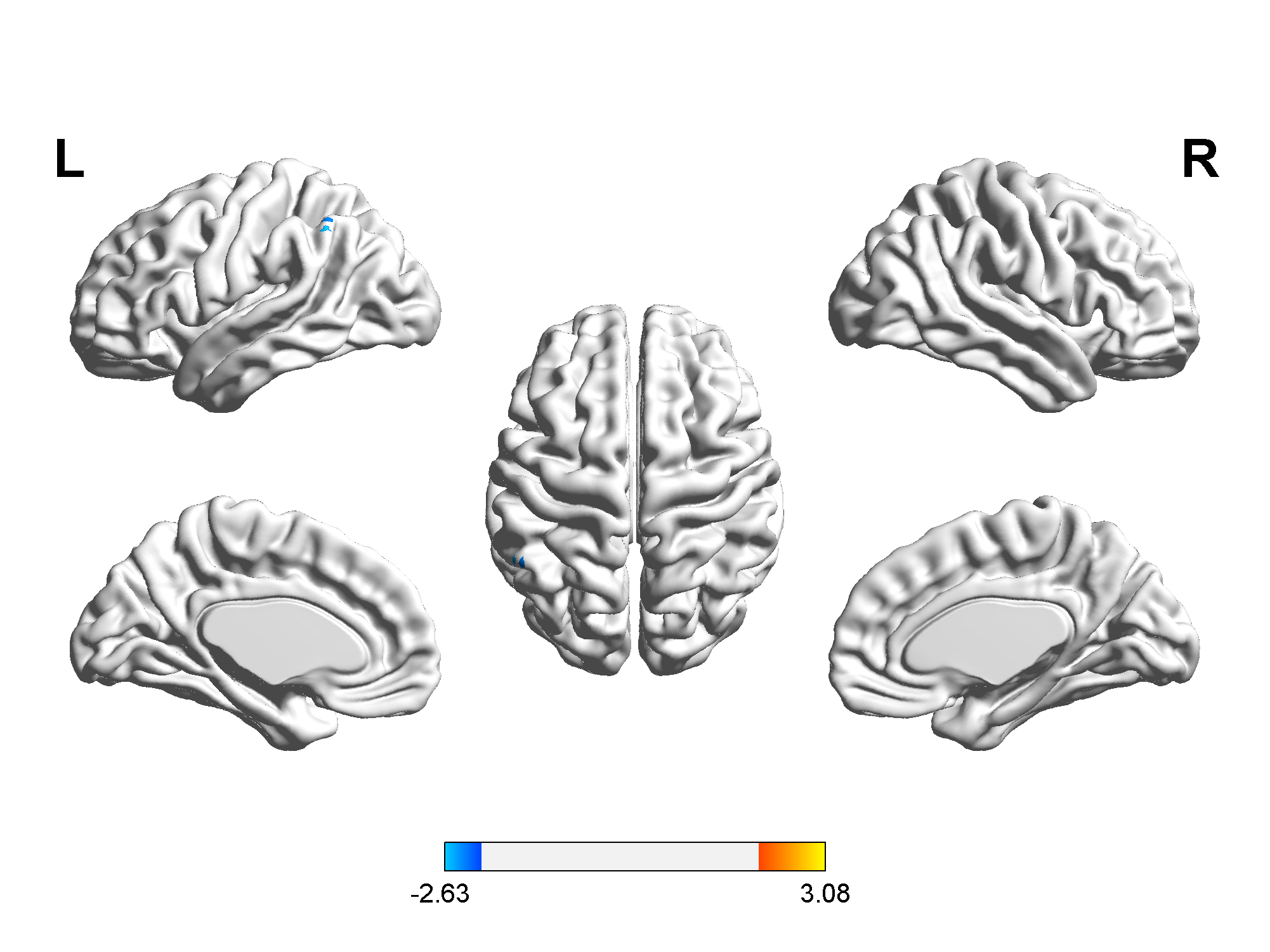

Supplement: Supplementary file 11 [file Image_3.TIF]

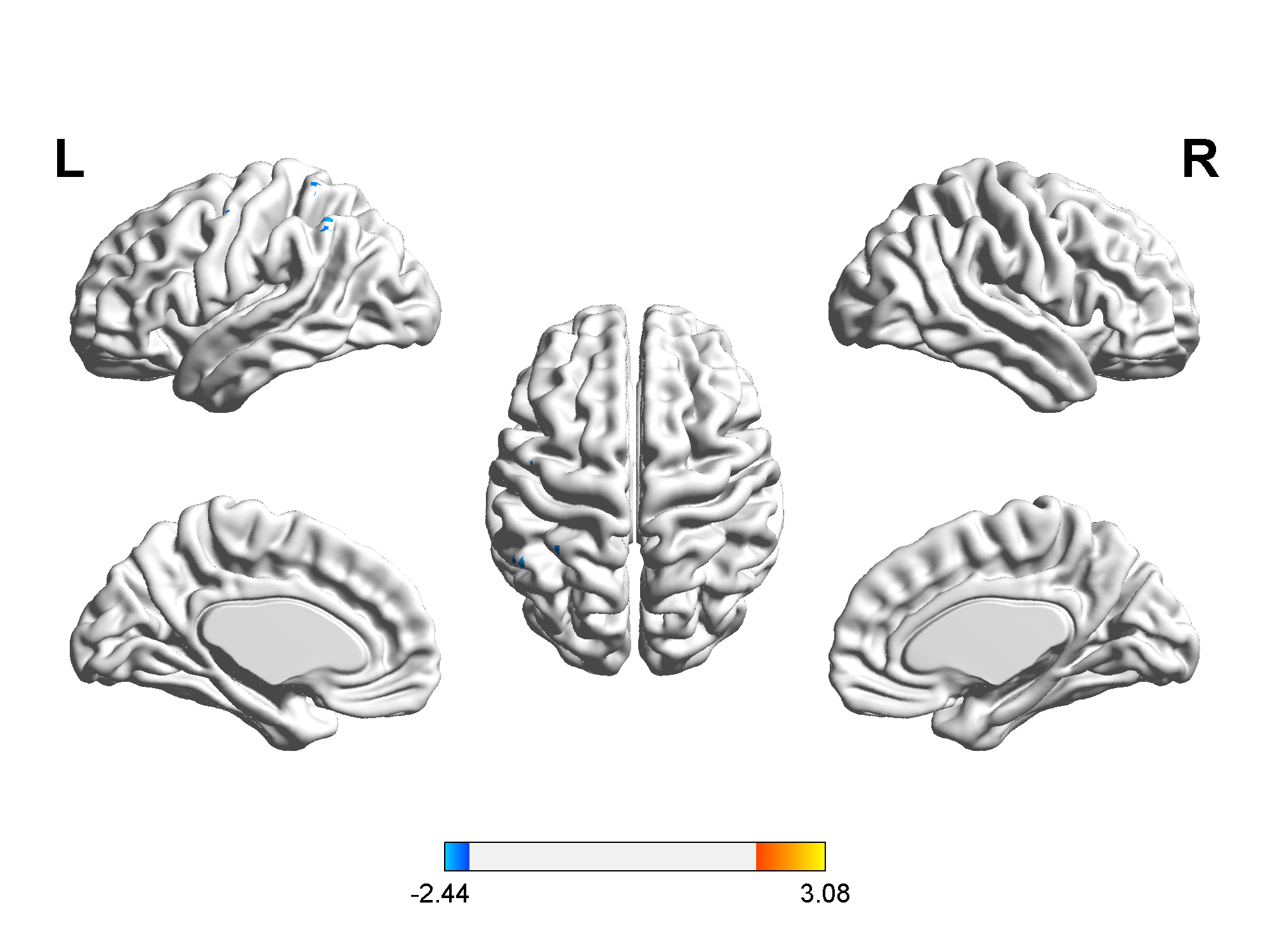

Supplement: Supplementary file 12 [file Image_4.TIF]
